# Digital learning designs in occupational therapy education: a scoping review

**DOI:** 10.1186/s12909-022-03955-x

**Published:** 2023-01-05

**Authors:** Na-Kyoung Hwang, Sun-Hwa Shim, Hye-Won Cheon

**Affiliations:** 1 Department of Occupational Therapy, Seoul North Municipal Hospital, 38, Yangwonyeok-ro, Jungnang-gu, Seoul, 02062 Republic of Korea; 2 Department of Occupational Therapy, College of Medical Science, Jeonju University, 303, Cheonjam-ro, Wansan-gu, Jeonju, Jeollabuk-do 55069 Republic of Korea; 3 Department of Dental Hygiene, College of Health Science, Howon University, 64, Howondae 3-gil, Impi-myeon, Gunsan, Jeollabuk-do 54058 Republic of Korea

**Keywords:** Digital learning design, Occupational therapy education, Learning technology, Learning outcomes

## Abstract

**Background:**

Digital learning is a rapidly advancing method for teaching and learning in professional health education. Although various digital learning designs have been tried in OT education, studies on digital learning designs are still limited.

**Methods:**

We conducted a scoping study that aimed to identify the digital learning designs used in occupational therapy (OT) education and review the effectiveness, learner perceptions, clinical skills integrated, and technology-based learning strategies used to facilitate learning. Four databases were searched using subheadings and terms relating to digital learning, occupational therapy, and education. The included studies were mapped according to the types of digital learning design, subjects, key clinical skills, and outcomes.

**Results:**

Twenty-two studies were included in this review, most of which were qualitative, observational, or mixed studies of the two designs. The digital learning designs identified in OT education were flipped, blended, hybrid, and distance learning, including e-learning and massive open online courses (MOOC). Among the components of clinical skills, professional reasoning and procedural knowledge were the most integrated into digital learning, and covered various OT subjects. Digital learning designs were reported to be equivalent to or more effective than the traditional face-to-face (F2F) class in learning outcomes of knowledge and skill acquisition, enhancing learning participation, reflection, and collaboration between learners. Various technologies have been used to promote synchronous or asynchronous active learning, providing learning strategies such as thinking, reflection, discussion, peer learning-group activity, and gamifying online learning.

**Conclusions:**

In OT digital learning, appropriate learning subjects, the arrangement of clinical skill components that can be well integrated into digital learning, and the selection of appropriate technologies for effective learning are important. The results should be confirmed within an experimental study design.

## Background

Digitalization has become a new opportunity and challenge for higher education today, and many educators and learners are participating in educational activities involving digitalization. In healthcare education, the learning space is expanding from the campus and clinical settings, which have been the main learning sites, to a virtual, digitalized space [1].

Digital learning is a popular and rapidly advancing learning method for teaching and learning in professional health education. It provides learning content to improve individual learners’ knowledge and skills and effective teaching methods through a variety of modalities using information and communication technologies such as computer-assisted, mobile, and digital simulation-based learning [2, 3]. Digital learning consists of digital teaching materials (e-textbooks, digital data, and content provided in digital format), digital tools (computers and smart devices), and digital delivery (Internet), which are provided in an integrated manner [4].

Digital learning design can be largely divided into blended and distance learning, such as a full e-learning course. Blended learning is a learning design that combines face-to-face (F2F) and online teaching with synchronous learning (provided in real-time, F2F or online) and asynchronous learning (provided in flexible time and online). Distance learning is a completely online learning design. In distance learning, learning and teaching take place using computers via a web-based system or a specific course management system that facilitates learner-teacher communication and is delivered completely asynchronously [5].

The professional occupational therapy (OT) program fosters culturally sensitive and evidence-based clinical competency by allowing learners to participate actively in the collaborative process between students, clients, and educators. Therefore, learners should be able to integrate academic knowledge, professional reasoning, and self-reflection through active learning through various experiences both inside and outside the classroom [6, 7]. Digital learning design in OT and physical therapy (PT) education has not been based on theoretical learning and has been frequently adjusted from a short perspective [1], although Bajpai et al. [8]. suggested guidelines for the theory of digital learning in professional health education. Nevertheless, various digital technologies (e.g., quizzes, videos, and social media) are currently being applied in the context of learning and teaching, such as learning feedback, assessment, clinical skills and techniques, and fieldwork supervision in OT and PT education [9].

Previous reviews have suggested that the effectiveness of blended learning [10], flipped learning [11], and e-learning [12] in healthcare education is equivalent or superior to traditional class teaching methods. Ødegaard et al. [13] also reported that blended learning and distance learning in PT education are equally or more effective than traditional teaching methods. In terms of planning digital learning in OT education, it is necessary to determine how to design digital learning to achieve learning outcomes and what clinical skills and subjects can be integrated into digital learning. Studies have applied various digital learning designs in OT education. However, a recent review of digital learning design has not been conducted, so it is necessary to explore the digital learning design studies conducted so far in OT education and to map and summarize the evidence for the applied digital learning design. This scoping review aims to identify (i) the digital learning design used in OT education, (ii) key clinical skills and subjects integrated into the design, (iii) technology-based learning strategies used to facilitate learning, and (iv) to explore digital learning outcomes and students’ perceptions.

## Methods

We adopt a scoping review methodology based on the process outlined by Arksey and O’Malley [14, 15]. In steps 2 and 3, the preferred reporting items for systematic reviews and meta-analyses (PRISMA) [16] were used to identify and select relevant studies.

### Stage 1: identifying the research question

To explore the literature on digital learning in OT education, we present the following research question: What digital learning design was applied in OT education, and what were the outcomes and students’ perceptions?

### Stage 2: identifying relevant studies

**Fig. 1:**
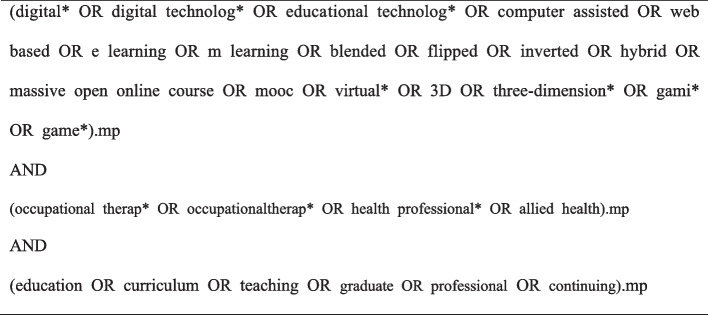
Search strategy for Medline Complete

Applicable research terms and database identifications were included to identify the relevant studies. The data search included Medline Complete, Embase, CINAHL, Scopus, and an additional search of grey literature using Google and Google Scholar. We also conducted a target-hand search of discipline-specific journals. These journals include the American Journal of Occupational Therapy, Journal of Occupational Therapy Education, Open Journal of Occupational Therapy, Journal of Physical Therapy Education, Health Professions Education, and Journal of Allied Health. Our basic search included keywords related to ‘digital learning’, ‘occupational therapy’, and ‘education’. Figure 1 shows an example of a search strategy.

### Stage 3: study selection

We included quantitative, qualitative, or mixed method study designs, full-text articles, English language, and material that met the following criteria for facilitating learning: those focused on digital learning design (e.g., blended learning, distance learning) or the use of technology-based learning strategies (e.g., peer learning group work, gamify online learning); a study population of OT students in bachelor’s/undergraduate, MOT (Master of Occupational Therapy), OTD (Occupational Therapy Doctorate), and occupational therapists participating in OT continuing education programs and reported on the outcomes of exams on knowledge and skills, usefulness, and students’ perceptions (e.g., satisfaction with learning, self-efficacy). In addition, the publication period was limited to January 2000 – February 2022. In the late 1990s, learning opportunities and designs using technologies such as the Internet, computers, e-mail, and interactive video and audio communication began to emerge as new educational models. Since the application of digital learning using technologies was limited before 2000 in OT education, the search was limited to studies published after 2000. The exclusion criteria were protocol studies, expert opinion studies, theses, dissertations, conference abstracts, education not for OT students or occupational therapists, and studies in which digital learning technologies were not used as part of a learning strategy. The authors agreed to include only studies that explicitly used digital learning designs in this scoping review through an iterative review process at the time of full-text review.

### Stages 4 and 5. Charting the data and collating, summarizing, and reporting the results

Two researchers independently extracted data from the included studies using an extraction form. Another researcher confirmed that the data synthesis strategy was followed and included data on the author, publication date, study design, participants, subject, key clinical skill and context, interventions (digital learning designs), outcomes (e.g., academic performance, participation, satisfaction), and student experience.

Clinical skills included physical examination skills, practical procedures, communication skills, and management. It also comprises basic scientific knowledge, procedural knowledge, and professional reasoning [17]. In this review, we identified the key clinical skills integrated into each digital learning design.

Several terms have been used for digital learning. In this review, digital learning types were classified according to the most frequently used terms in the literature pertaining to the technical and pedagogical aspects of educational technology [13] and previous reviews on digital learning designs in physiotherapy education [18].

#### Blended learning

Blended learning refers to a mixed system of education involving the mobilization of learning contexts such as face-to-face and online learning. It also focuses on the integration of different teaching methods, the interaction of different technological tools, and the adoption of virtual spaces in the educational process [18]. Mixed learning by adding online learning materials and activities to offline classes is not intended to replace traditional F2F classes [19, 20]. Examples include a scene in which online activities, such as communication and sharing activities using tablets and smartphones, or realistic content, such as augmented reality (AR), are integrated into offline classes.

#### Hybrid learning

Some students attend classes in person, whereas others attend classes virtually at the location of their choice. Educators use tools such as video-conferencing hardware and software to teach remote and F2F students simultaneously. Some F2F classes are replaced by online components, and online interactions can be either synchronous (e.g., online interactions in real-time via Zoom) or asynchronous (e.g., online interactions at different times via online discussions or VoiceThread) [19, 21].

#### Flipped learning

The method of interaction between students and the learning content in traditional classes is reversed. A method of learning that typically includes both F2F and online components in which basic knowledge is learned prior to class, such as reading, podcasts, or videos, and then expanded through activities conducted in class with the support of an instructor [19, 22].

#### E-learning

This is a form of distance learning that allows learners access from different geographic locations. Various elements of the education strategy (e.g., animations, graphics, videos, forums, chats, quizzes) are delivered in an electronically structured course. Students and instructors can use e-learning systems both asynchronously and synchronously. Synchronous e-learning can be conducted in a real-time interactive manner, and even when simultaneous online access is not possible, forums, e-mails, and mailing lists can support student-instructor relationships, enabling flexible learning [23].

#### M-learning

A form of e-learning that has emerged with the use of mobile devices in education, typically used outside the classroom. People can use their mobile devices to access educational resources, connect with others, or create content inside and outside the classroom [23].

#### Massive open online courses (MOOC)

“MOOC integrates the connectivity of social networking, the facilitation of an acknowledged expert in a field of study, and a collection of freely accessible online resources” [24]. The course includes videos, exercises, presentations, and assessments.

The thematic information identified and extracted from each study was tabulated based on the type of digital learning design, integrated subject, context, comparison group, detailed learning activities, and key findings. Textual descriptions were created after analysis according to the digital design type. The themes and summaries of the studies were organized by research question (Table 1), and findings related to each question were discussed.

**Table 1:** Characteristics of the included studies

Author year	Study design	Participants	Learning design, Subject, Context
Barillas 2019 [25]	Quasi-experimental	MOT program, 1st year students (*n* = 35)	**Learning design:** Blended learning **Subject:** human anatomy **Clinical skills:** basic science knowledge **Context:** on campus
Howard 2019 [26]	Mixed	OTD and MOT program, 1st year students (*n* = 74)	**Learning design:** Blended learning **Subject:** OT theory, FOR **Clinical skills:** procedural knowledge **Context:** on campus
Simons et al.2002 [27]	Mixed	MOT program, 1st year students (*n* = 19) & Teacher education graduate school students (*n* = 31)	**Learning design:** Blended learning **Subject** OT course: OT theories, principles Teacher education course: reading in the content areas **Clinical skills:** procedural knowledge **Context:** on campus
Grant 2019 [28]	Mixed	OT undergraduate, 2nd year students (*n* = 42)	**Learning design:** Blended learning **Subject:** exploring the adaptive equipment and developing skills to use **Clinical skills:** procedural knowledge **Context:** on campus
Lin et al. 2021 [29]	Mixed	OT undergraduate, 3rd year students (n = 42)	**Learning design:** E- learning **Subject:** psychosocial dysfunction **Clinical skills:** procedural knowledge **Context:** on campus
Power et al. 2020 [30]	RCT	OT undergraduate, 1st year students (*n* = 30)	**Learning design:** E- learning **Subject:** SCA-based CPT **Clinical skills:** procedural knowledge **Context:** on campus
Carbonaro et al. 2008 [31]	Mixed	Health science undergraduate program students: medicine, nursing, pharmacy, OT, PT, dentistry, dental hygiene, medical laboratory science, and nutrition (*n* = 49)	**Learning design:** Blended learning **Subject:** interprofessional team process skills **Clinical skills:** procedural knowledge **Context:** on campus
Martín-Valero et al. 2021 [32]	Quasi-experimental	OT and PT undergraduate, 2nd–4th year students (*n* = 138)	**Learning design:** MOOC **Subject:** support products, ergonomics and autonomy, personal autonomy in mental health, psychopathology in mental health, activity analysis, pain and hospitalization **Clinical skills:** procedural knowledge, professional reasoning **Context:** on campus
Henderson et al. 2020 [33]	Mixed	MOT program, 2nd year students (*n* = 43)	**Learning design:** Flipped classroom **Subject:** adult practice **Clinical skills:** procedural knowledge, professional reasoning **Context:** on campus
Jedlicka et al. 2002 [34]	Mixed	OT undergraduate (*n* = 22)	**Learning design:** E- learning **Subject:** mental health programming **Clinical skills:** procedural knowledge, professional reasoning **Context:** on campus
Thomas et al. 2005 [35]	Qualitative	OT undergraduate, 1st year students (n = 42)	**Learning design:** E- learning **Subject:** OT fieldwork education **Clinical skills:** procedural knowledge, professional reasoning **Context:** on fieldwork placement
Myers et al. 2015 [36]	Qualitative	MOT, MSLP program, 2nd year students & DPT program, 3rd year students (n = unspecified)	**Learning design:** E- learning **Subject:** interprofessional skills on early childhood practice and school-based practice **Clinical skills:** procedural knowledge, professional reasoning **Context:** on clinical setting
Kim et al. 2022 [37] Mixed	Mixed	Occupational therapist (n = 43)	**Learning design:** E- learning **Subject:** DLW framework **Clinical skills:** procedural knowledge, professional reasoning **Context:** on clinical setting
Barnard-Ashton et al. 2017 [38]	Qualitative	OT undergraduate (*n* = 1000) & lecturers (n = 9)	**Learning design:** Blended learning **Subject:** PBL scenarios on OT undergraduate program **Clinical skills:** procedural knowledge, professional reasoning **Context:** on campus
Murphy et al. 2018 [39]	Quasi-experimental	Integrated BS*/*MOT 2nd year students (*n* = 61)	**Learning design:** Blended learning **Subject:** case-based professional reasoning **Clinical skills:** professional reasoning **Context:** on campus
Gee et al. 2017 [40]	Descriptive	MOT program, 1st year students (*n* = 12)	**Learning design:** E-learning course **Subject:** sensory processing **Clinical skills:** professional reasoning **Context:** on campus
Mitchell et al.2009 [41]	Descriptive	MOT program, 1st-year students (*n* = 21)	**Learning design:** E-learning course **Subject:** case application of the OPPM **Clinical skills:** professional reasoning **Context:** on campus
Feldhacker et al. 2022 [42]	Mixed	OTD program, 2nd year students (*n* = 116)	**Learning design:** Hybrid learning **Subject:** all OTD courses **Clinical skills:** unspecified **Context:** on campus
Banning et al. 2021 [43]	Analytical	OTD program, graduate (*n* = 168)	**Learning design:** Hybrid learning **Subject:** all OTD courses **Clinical skills:** unspecified **Context:** on campus
Lewis-Kipkulei et al. 2021 [44]	Qualitative	OTD program & SPED undergraduate students (n = 13)	**Learning design:** Flipped classroom **Subject:** some courses **Clinical skills:** unspecified **Context:** on campus
Benaroya et al. 2021 [45]	Descriptive	OTA students (*n* = 20)	**Learning design:** E- learning **Subject:** 9 OTA courses (e.g. history of OT, mental health and wellness, pediatrics) **Clinical skills:** unspecified **Context:** on campus
Provident et al. 2015 [46]	Qualitative	OTD program, graduates (*n* = 113)	**Learning design:** E- learning **Subject:** all OTD courses **Clinical skills:** unspecified **Context:** on campus

*RCT* randomized controlled trial, *MOT* Master of Occupational Therapy, *OPPM* occupational performance process model, *OTD* Occupational Therapy Doctorate, *BS* bachelor’s degree, *MOOC* massive open online courses, *OT* occupational therapy, *PT* physical therapy, *MSLP* Master of Speech and Language Pathology, *DPT* Doctor of Physical Therapy*, FOR* frames of reference, *SCA* supported conversation for adults with aphasia, *CPT* communication partner training, *OTA* Occupational Therapy Assistant, *SPED* special education, *PBL* problem-based learning, *DLW* do-live-well

## Results

### General features of the selected studies

**Fig. 2:**
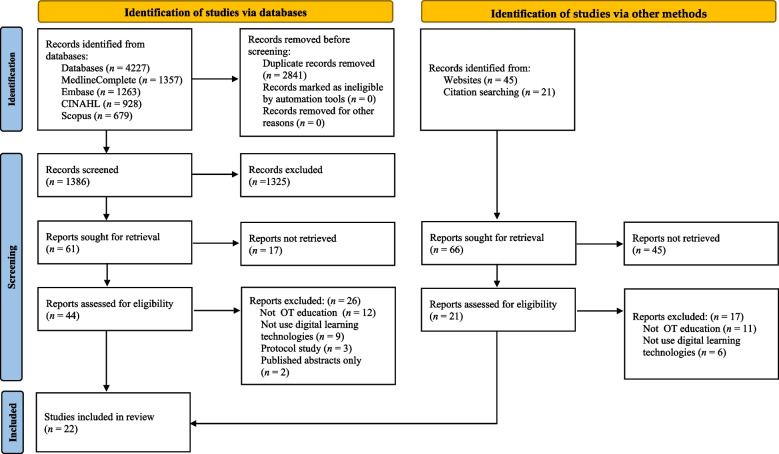
The Preferred Reporting Items for Systematic Reviews and Meta-Analyses (PRISMA) flow chart. OT: occupational therapy

We included 22 studies (Fig. 2) with 2143 participants (sample size range between mi*n* = 10; max = 1009). The participants were occupational therapists (*n* = 1), OTD program students (*n* = 5), MOT program students (*n* = 7), integrated BS/MS OT program students (*n* = 1), OT undergraduate students (*n* = 8), and the occupational therapy assistant (OTA) program (n = 1). In addition to students majoring in OT, students majoring in physical therapy, speech and language pathology, medicine, nursing, dentistry, dental hygiene, and nutrition were also included in the studies. The study design was used to investigate the effects of digital learning on academic performance and participants’ perceptions of digital learning experiences. Mixed methods (*n* = 9) were the most common, followed by qualitative methods (gathered interview and focus group data) (*n* = 5), quasi-experimental (*n* = 3), randomized controlled trial (*n* = 1), descriptive (n = 3), and analytical (n = 1) methods. A summary of the characteristics of the selected studies is shown in Table 1.

### Key clinical skills, subjects, and outcomes

The identified key clinical skills components included basic scientific knowledge (*n* = 1), procedural knowledge (*n* = 6), professional reasoning (*n* = 3), and combined procedural knowledge and professional reasoning (*n* = 7). One study included a basic science knowledge component, and 3D anatomy software was used for the anatomy course [25]. Studies that included procedural knowledge components addressed theories, frames of reference [26, 27], adaptive equipment [28], psychosocial dysfunction [29], communication partner training for adult aphasia [30], and interprofessional team skills [31]. Studies involving both procedural knowledge and professional reasoning addressed ergonomics, autonomy, activity analysis, and other OT subjects [32], adult practice [33], mental health [34], fieldwork training [35], interprofessional team skills in childhood, school practice [36], do-live-well framework [37], and problem-based learning (OTA:) scenarios [38]. In addition, the studies included only professional reasoning elements and provided case-based professional reasoning training, such as sensory processing and occupational performance process model (OPPM) application of cases [39–41] (Table 1).

The variables used to measure the outcomes of digital learning were academic performance (e.g., course grades, exam scores, course objective achievement) (*n* = 11), professional reasoning skills (*n* = 4), class participation (*n* = 3), satisfaction with learning (*n* = 2), preference for instructional methods (n = 2), usefulness (n = 2), cognitive and emotional empathy (*n* = 1), a sense of belonging, acquired skills, practice setting, and leadership (n = 1), metacognition of learning (n = 1), and self-efficacy with computer technologies (n = 1). In addition, students reported the benefits and challenges they perceived in digital learning, such as activation of interaction, satisfaction, improved self-confidence, increased participation, effectiveness and efficiency, promotion of active learning, and improvement of professional reasoning and busywork (*n* = 13) (Table 2).

**Table 2:** Summary of intervention and results of the included studies

Author Year	Intervention	Results
Barillas 2019 [25]	**Experimental group:** 3D anatomy software + on-campus class (4-hour lecture, 4-hour laboratory time) **Control group:** taken a course without use of anatomy software **Duration:** 1 semester	**Course grades:** higher overall final course grades in experimental group compared to the control group, but not statistically significant (*p* > 0.05); no statistically significant differences in lecture and laboratory exam grades between two groups (*p* = 0.891, *p* = 0.507, respectively) **Usefulness:** 82 of students reported the use of the anatomy software to be helpful in understanding course concepts. **Course grade differences:** no statistically significant among the different learning styles or age groups (*p* > 0.05)
Howard 2019 [26]	**Blended group:** F2F sessions (lectures, lab activities) + online sessions (videos, reading assignments, group work, online forum) via LMS **F2F group:** traditional lecture and lab, with learning activities **Duration:** 1 semester	**Summative course grades:** statistically significantly higher in F2F group **Qualitative analysis** - Students’ perception on online hybrid section: the course required more busywork. - Priori themes: value of theory and growth in theory application - Emergent themes: use of theory for professional reasoning, client-centered practice, theory integration in practice, and theory in the OT process
Simons et al. 2002 [27]	**Experimental group:** F2F sessions + online sessions (lectures, presentations, web sites links, and discussion, assignments) via LMS; Web-Course-In-a-Box **Duration:** 1 semester	**Online learning experience:** students reported that it was efficient, effective, and satisfactory, as previously expected. **ASCT:** increased significantly during the semester only in OT course (word processing: *p =* 0.000, e-mail: *p =* 0*.*013, use of the Web: *p =* 0.025) **Qualitative analysis** - Efficiency, effectiveness, and satisfaction of online learning are a product of the interaction between the individual student factors, remote learning environment factors, and course design and instructor factors.
Grant 2019 [28]	**Experimental group:** Game-based technology during a class to encourage the engagement with practical aspects of equipment use and fitting, Individual and group game missions **Duration:** 3-hour	**Students’ perception of game-based learning** - Extremely high student engagement and increased confidence in practicing using adaptive equipment - Students perceived that the game-based technology is useful tool to get knowledge about adaptive equipment. **Qualitative analysis** - Students mentioned that game-based learning allowed them to experience autonomy and competition.
Lin et al. 2021 [29]	**Smart class group:** online teaching information platform ; pre-class (requirements, studying materials), in-class (theoretical class: discussion, questions, and answers/ practical class: practice and discussion) after- class (assignments, group discussion, extracurricular studying resources) **Traditional class grou**p: F2F teaching (theoretical class + practical class) **Duration:** 1 semester	**Course scores:** no significant difference in final score between two groups (*p* = 0.874); higher assignment score and practical exam score in the smart class group than those in the traditional class group (*p* = 0.017, *p* = 0.040, respectively) **Pre-class preview, in-class learning, after-class review:** no significant difference between two groups (*p* > 0.05, all) **Course satisfaction:** no significant difference between two groups (*p* > 0.05) **Students’ questionnaire survey and teachers’ interview:** preference for combining the two learning methods (smart class module for the theory class + traditional class for the practical class)
Power et al. 2020 [30]	**Online group:** online CPT program at the computer lab on campus (text and audio information, video demonstration for SCA training) **F2F group:** CPT training from a presenter at the standard lecture theatre **Control group:** attended lectures not related to this topic and, completed the online CPT program after the study **Duration:** 45-min	**AASK** - Compared with the control group, significantly higher knowledge of aphasia in online and F2F groups (online vs. control: *p* = 0.000; F2F vs. control: *p* = 0.002), knowledge of facilitative strategies (online vs. control: *p* = 0.000; F2F vs. control: *p* = 0.002), and positive attitudes towards aphasia (online vs. control: *p* = 0.031; F2F versus control: *p* = 0.032) - No significant difference between the online and F2F groups for the total or any subtotals (*p* = 1.000)
Carbonaro et al. 2008 [31]	**F2F group:** student manual and interprofessional team discussion on complex case scenarios **Blended group: F2F** sessions + online sessions (synchronous virtual classroom and asynchronous interactions; library resources, video clips, download notes and assignments, submit work via LMS) **Duration:** 5 weeks	**Interprofessional team attitudes, knowledge, and skill:** no significant differences in communication and teamwork, interprofessional relationship, interprofessional learning, interprofessional interaction between groups (*p* = 0.376, *p* = 0.769, *p* = 0.174, *p* = 0.462, respectively) **Team dynamic:** no observable differences between the groups; comparable performance before and after global rating and overall competence in the groups **Qualitative analysis** - Achievement of course learning objectives perceived by students: a more positive achievement in the blended learning class (collaborating effectively as a healthcare member)
Martín-Valero et al. 2021 [32]	**Experimental group:** university training with MOOC (videos for brief theoretical explanation, clinical cases, and discussion forums for analysis, professional reasoning) **Control group:** only university training without MOOC **Duration:** unspecified	**Academic performance:** statistically significant differences between the groups in favor of the MOOC **Evaluation rubric after MOOC:** quite good result in MOOC group (X = 21; SD = 6.88, out of a maximum of 36 points) **MOOC and empathy:** statistically significant differences between the pre and post empathy scores (X = − 11.71; SD = 11.36; t (173) = − 13.68, *p* < 0.001) with a high effect size (d = 0.86) **Structural equation model:** the higher the realization and participation in MOOC, the higher the academic performance, and cognitive and affective empathy
Henderson et al. 2020 [33]	**Flipped course group**: pre-class activities (online lectures, readings, learning activity via online discussion board, etc.) + in-class activities (role playing, case studies, and collaborative group work for active application of learned knowledge) **Collaborative group:** involved in course design; meeting weekly with the researcher for creation of feedback, comments and opinions on the flipped course **Duration:** 1 semester	**APLOS:** significant growth in both groups, no differences between the groups at post-course **SACRR:** similar professional reasoning skills in both groups at post-course, no significant differences (Hotelling’s T, F = 1.240, *p* < 0.333) **PMQ:** significant differences (Hotelling’s T, F = + 2.745, *p* < 0.011) on 3 of 18 items; the collaborative group showed more metacognitive learning characteristics. **Qualitative analysis** - Experienced growth in active learning, professional reasoning, and change in student engagement in both groups - Additional benefits of development of relationships, increased accountability, and improved metacognitive learning in collaborative group
Jedlicka et al. 2002 [34]	**Experimental group:** applying and synthesizing lecture content to cases while rotating 3 online educational methods **-** Two-way interactive video and audio group - Chat room group - Independent learning group: independent case assignments - Three groups: lectures, discussions, and lab experiences using two-way interaction LMS; WebCT before case application training **Duration:** 1 semester	**Exam of student’s performance:** no significant difference between three groups (*p* = 0.11) **Student preference for instructional methods:** 77% of students prefer two-way interactive video and audio **Qualitative analysis** - Activation of interaction and communication between learners is important for effective education through distance learning. - Higher skills of faculty members for various skills are required.
Thomas et al. 2005 [35]	**Experimental group**: online fieldwork education via LMS; WebCT - Post messages (clinical setting, OT roles, client case history) on bulletin board by students and participating in virtual discussions - Monitoring discussion boards by instructors **Duration:** 6 weeks	**Participation** - 95% (40/42) general login to the discussion board - 92.5% (37/40) of the participating students actually read the postings - 87.5% (35/40) posted at least 1 item on the bulletin board **Qualitative analysis** - Majority of the students’ postings: knowledge, comprehension, application - Beneficial effects of participation in WebCT during fieldwork: student learning and achievement of stage 1 learning objectives by supporting students in peer learning, improving student autonomy, supporting self-directed learning and stimulating higherorder thinking
Myers et al. 2015 [36]	**Experimental group**: online interprofessional skill training via LMS; Blackboard - Case study assignments, blogs for online discussions, and multimedia content (web resources, videos, and peer-reviewed literature) - Small group activities (development of goals, intervention plans, and recommendations for caregivers and school personnel) **Duration:** 1 semester	**Qualitative analysis** - Students perceived that their understanding and knowledge of other disciplines’ roles on the team increased. - Students felt more comfortable with the idea of collaborating with other disciplines. - Problem solving and decision-making were improved through factual knowledge about content and the process of applying it. - Students recognized critically analyzing and evaluating viewpoints as the most positive aspect of learning through this course. **Use of multimedia resources**: highly rated in course feedback
Kim et al. 2022 [37]	**F2F group:** 4-session lectures on DLW and wrap-up + small group discussion on video case scenarios, single-day **Online group:** prerecorded DLW sessions + asynchronous discussion forums (same content as F2F delivered) **Duration:** 8-hour	**Knowledge changes regarding the DLW framework:** no significant differences in at the 3 time points; pre, post, follow-up between the groups (*p* = 0.57 to *p* = 0.99) **Factors influencing DLW adoption:** significant differences between the groups at posttest in favor of the F2F group (*p* = 0.001) **Satisfaction with the workshop**: significant differences between the groups at posttest in favor of the F2F group (*p* < 0.001) **Qualitative analysis** - Relevance to their practices and interests may improve learning, a familiar learning environment may facilitate learning. - F2F workshop is valuable in the learning process. - Flexibility in web-based learning can be both beneficial and challenging; participants expressed web-based learning lacked in-person-like interactions.
Barnard-Ashton et al., 2017 [38]	**Experimental group**: F2F sessions (problem scenario introduction, mid-problem tutorials, and problem feedback) + online activities (podcasts, online discussion forums, live video calling, open source VLE, etc) **Duration:** 1 semester course for 6 years	**Qualitative analysis** - Lecturers and students noted improved communication, curriculum transparency and efficient use of time and paper resources, which in turn accommodates the student’s need for instant gratification. - Students felt reassured that they had the correct information and any-time access to all communication resulting in the use of blended learning becoming part of their study habits. - Lecturers perceived success in seeing students actively engaged with blended learning activities and evidence that blended learning was contributing to improved pass rates.
Murphy et al. 2018 [39]	**Experimental group:** online videos of cases in real clinical setting and student discussion on professional reasoning in class, out-of-class assignments (small group and individual) **Control group:** written case studies using a textbook and student discussion on professional reasoning in class, text-based case study assignments (small group and individual) **Duration:** 1 semester	**HSRT** - Experimental group (pre-post): statistically significant changes in overall score, percentile, induction, deduction, and evaluation (*p* < 0.05); but, not statistically significant in analysis and inference - Control group (pre-post): no statistical significance in all items - Comparison: statistically significant difference in inductive reasoning skill (*p* = 0.03); but no significant difference in the other items
Gee et al. 2017 [40]	**Experimental group:** professional reasoning process using A SECRET module - Simulated case scenario via multimedia (video, audio, and text) - Multiple choice assessment related to each of the 7 elements of A SECRET via LMS **Duration:** 1 semester	**Evaluation of teaching professional reasoning process (A SECRET):** overall average score 68% in the strategy achievement, positive findings in novelty of the instruction, assessment, and the content
Mitchell et al. 2009 [41]	**Experimental group:** online independent study - Reading assignments about OPPM - Written objective examination - Submission of responses to case questions and feedback via LMS; Blackboard - Completion of critical reasoning journals **Duration:** 1 semester	**The effectiveness of online assignments on enhancing awareness and use of professional reasoning skills:** more than 40% of each type of reasoning on the WGCTA by students. **Students’ perceptions of the use of professional reasoning:** SR is the most used in each part of the assignment **Primary factors in using reasoning type:** the type of information being considered, the actions required by the question, and the student’s innate style and previous experiences
Feldhacker et al. 2022 [42]	**F2F group:** all lectures and labs on campus **Hybrid group:** recorded lectures and class sessions (synchronously or asynchronously) + only attended labs and experiential learning components on campus **-** Both group: use the active learning strategies in class sessions (group discussions, think-pair-share, polling, quizzes, reciprocal questioning, and others), online activities (Flipgrid, Padlet, online quizzes, and discussion posts). **Duration:** 1 semester	**Achievement of learning course objectives and acquisition of knowledge:** significant improvement in both groups from pre- to posttest (*p* < 0.05); no statistically significant differences between groups at pre- or posttest **Qualitative analysis** - Students reported the effectiveness of course design (active learning strategies) regardless of course delivery method and strongly favored assignments and learning activitieslinked to real-life experiences.
Banning et al. 2021 [43]	**Hybrid pathway:** F2F (47% of the total credit hours, on-campus and community-based labs, service-learning experiences, and fieldwork) + online delivery (53% of the total credit hours, online lectures, exams, and synchronous online delivery lab) **F2F pathway:** only F2F elements (lectures, labs, learning activities) **Duration:** unspecified	**Perceived preparedness for the certification exam or to enter the workforce:** no significant difference between the groups **Sense of belonging, skills learned throughout the program, practice settings or leadership roles held after graduation:** no significant differences between the groups **The number of state occupational therapy associations**: significantly greater number of hybrid alumni
Lewis-Kipkulei et al. 2021 [44]	**Experimental group:** pre-class (assigned readings, guiding questions, and research topics) + in-class (peer collaboration, student-led discussion, and peer teaching) **Duration:** 1 semester	**Qualitative analysis** - Flipped classroom has a positive impact on peer interaction and collaboration. - Flipped classroom provides students more individualized time. - The learning model had a positive impact on encouraging higher student engagement. - Learning through discussion was much more valuable than traditional lecture courses. - The course was more student-focused, supporting independent learning.
Benaroya et al. 2021 [45]	**Experimental group: OTA online education using active learning strategies** - Synchronous virtual classroom platform: flipped classroom, think-pair-share and jigsaw technique using breakout rooms, polling and student response systems, muddiest point via chat box, lab kits, - LMS: 1-minute paper using discussion forums, student-generated video **Duration:** 2 semesters	**Helpfulness of each strategy:** breakout room and chat box feature of the synchronous virtual classroom, lab kits were perceived as most helpful, whereas student-generated videos and one-minute papers were perceived as least helpful
Provident et al. 2015 [46]	**Experimental group:** online OTD program via LMS; Moodle - Assignments related to each student’s professional interest/practice - Ongoing discussion forums by instructors - Reflective writing activities and peer review processes - Implementation of the capstone project - 2 campus visits for leadership course, presentation of capstone projects **Duration:** 4 semesters	**Qualitative analysis** -Students had multiple opportunities for critical reflection and discourse throughout the program; students experienced professional uneasiness or a dilemma. - Students reflected that the program’s cohort structure allows for the sharing and interaction of experiences with instructors and other students. - Capstone projects provided active learning in each student’s unique worksite making the personal transformation more evident to the student. - Students reported increased confidence in their new roles and increased awareness of positive change after completing the OTD program.

*3D* three-dimensional, *F2F* face to face, *OT* occupational therapy, *ASCT* adapted self-efficacy with computer technologies, *AASK* aphasia attitudes, strategies and knowledge, *CPT* communication partner training, *SCA* supported conversation for adults with aphasia, *MOOC* massive open online courses, *APLOS* adult practice learning objectives survey, *SACRR* self-assessment of clinical reflection and reasoning, *PMQ* Plattner metacognition questionnaire, *WebCT* web courset tools, *DLW* do-live-well, *VLE* virtual learning environment, *HSRT* health science reasoning test, *A SECRET* attention, sensation, emotional regulation, culture/context/condition, relationships, environment, and task, *OPPM* occupational performance process model, *SR* scientific reasoning, *WGCTA* Watson-Glaser critical thinking appraisal, *OTA* Occupational Therapy Assistant, *OTD* Occupational Therapy Doctorate

### Digital learning design and outcomes

#### Blended learning

Blended learning was used in seven studies. Barillas [25] used 3D anatomy software with F2F sessions on human anatomy subjects; the blended group showed higher learning outcomes than the F2F group, and students reported that the software was helpful in understanding the course concept. Grant [28] used game software for the use and fitting of adaptive equipment during classes. The participants showed high participation in game-based learning and increased confidence in the practice of adaptive equipment. Other studies using blended learning have integrated synchronous or asynchronous online sessions with F2F sessions and addressed various course subjects. Howard [26] addressed OT theory: the F2F group showed significantly higher academic performance than the blended group, and the blended group reported that online sessions required a lot of busy work. However, in the study by Simons et al. [27], students reported that blended learning in OT theory was effective, efficient, and satisfactory, as expected before the course. Murphy et al. [39] addressed case-based professional reasoning; the blended group showed a significant improvement in overall reasoning in the pre-test and post-test, unlike the F2F group. In the study by Barnard-Ashton et al. [38], the subject was problem-based learning scenario lecturers, and students reported that blended learning facilitated active learning (improved communication and efficient use of time and learning resources). Carbonaro et al. [31] addressed interprofessional skills for undergraduate health science students composed of several majors. The blended and F2F groups showed similar improvements in learning outcomes, and the blended group students reported that the class had a positive impact on collaboration as healthcare members (Tables 1, 2).

### Hybrid learning

Hybrid learning was adopted in two studies. Feldhacker et al. [42] provided all OTD courses for one semester in two delivery types: hybrids and F2F. After completion of the course, both the hybrid and F2F groups showed similar improvements in learning outcomes, and students reported that tasks linked to real-life experiences facilitated active learning, regardless of course delivery type. In the study by Banning [43], the two groups also showed similar improvements in learning outcomes, and there was no significant difference in the perception of certification exams or job preparation (Tables 1, 2).

### Flipped classroom

A flipped classroom design was used in two studies. Henderson et al. [33] compared the flipped course group with the subject of adult practice and the group involved in the flipped course design; both groups showed equal effects on learning outcomes and professional reasoning skills. Students participating in the study by Lewis-Kipkulei et al. [44] mentioned that flipped learning has a positive impact on peer interaction and collaboration, and the benefits of having more personalized time for learning (Tables 1, 2).

### E-learning

Ten studies used e-learning courses. In three studies with a F2F comparison group, there were no significant differences between the e-learning and F2F groups in course satisfaction or academic performance. The course subjects of these studies were psychosocial dysfunction [29], the communication partner training (CPT) program for adults with aphasia [30], and the DLW framework [37], which were delivered by e-learning and F2F. Six studies included course subjects without the F2F comparison group. Gee et al. [40] addressed case-based professional reasoning for sensory processing, and students trained in the professional reasoning process showed high achievement in professional reasoning strategies and reported positive aspects in teaching methods, evaluation, and content. In the e-learning course, five studies used OT courses through the learning management system (LMS). Benaroya et al. [45] and Provident et al. [46] provided online courses using active learning strategies (e.g., discussion forums, reflective writing activities, and peer review) through an LMS. Students reported that the use of online platforms with integrated active learning strategies increased interaction and sharing between learners and helped them learn. In particular, it was reported that Provident et al.’s [46] capstone project led to personal transformation at each unique worksite. Thomas et al. [35] conducted online fieldwork training during placement via the LMS. Students showed a high participation rate (95%), commented on the advantages of active learning integrated into e-learning, such as peer learning, autonomy, and self-directed learning, and promoted higher-order thinking. Myers et al. [36] provided inter-professional skill training consisting of case study assignments, blogging, multimedia content, and small group activities via the LMS to students majoring in OT, PT, and speech-language pathology (SLP). Students perceived that this e-learning course improved their understanding and knowledge of different disciplines’ roles and was useful for developing critical analysis and evaluation skills from the viewpoint of problem-solving. Mitchell et al. [41] also used an active learning strategy through LMS and reported that online tasks had a positive effect on reinforcing awareness and the use of professional reasoning skills. One study compared three online delivery methods [34]. The study was conducted by rotating three online methods: two-way interactive video and audio, chat room groups, and independent case assignments. There was no significant difference in students’ task performance between the three methods, and students reported that interaction between learners was an important factor for effective distance learning (Tables 1, 2).

### MOOC

Only one study provided MOOC [32], videos for brief theoretical explanations, clinical cases, and discussion forums for analysis, and professional reasoning was provided to various OT subjects (e.g., support products, ergonomics, and autonomy). The MOOC group showed a high participation rate in learning and a significant difference in cognitive and affective empathy scores before and after the tests and showed higher academic performance compared to the control group that provided only the undergraduate program (Tables 1, 2).

### Technologies used to promote active learning

**Fig. 3:**
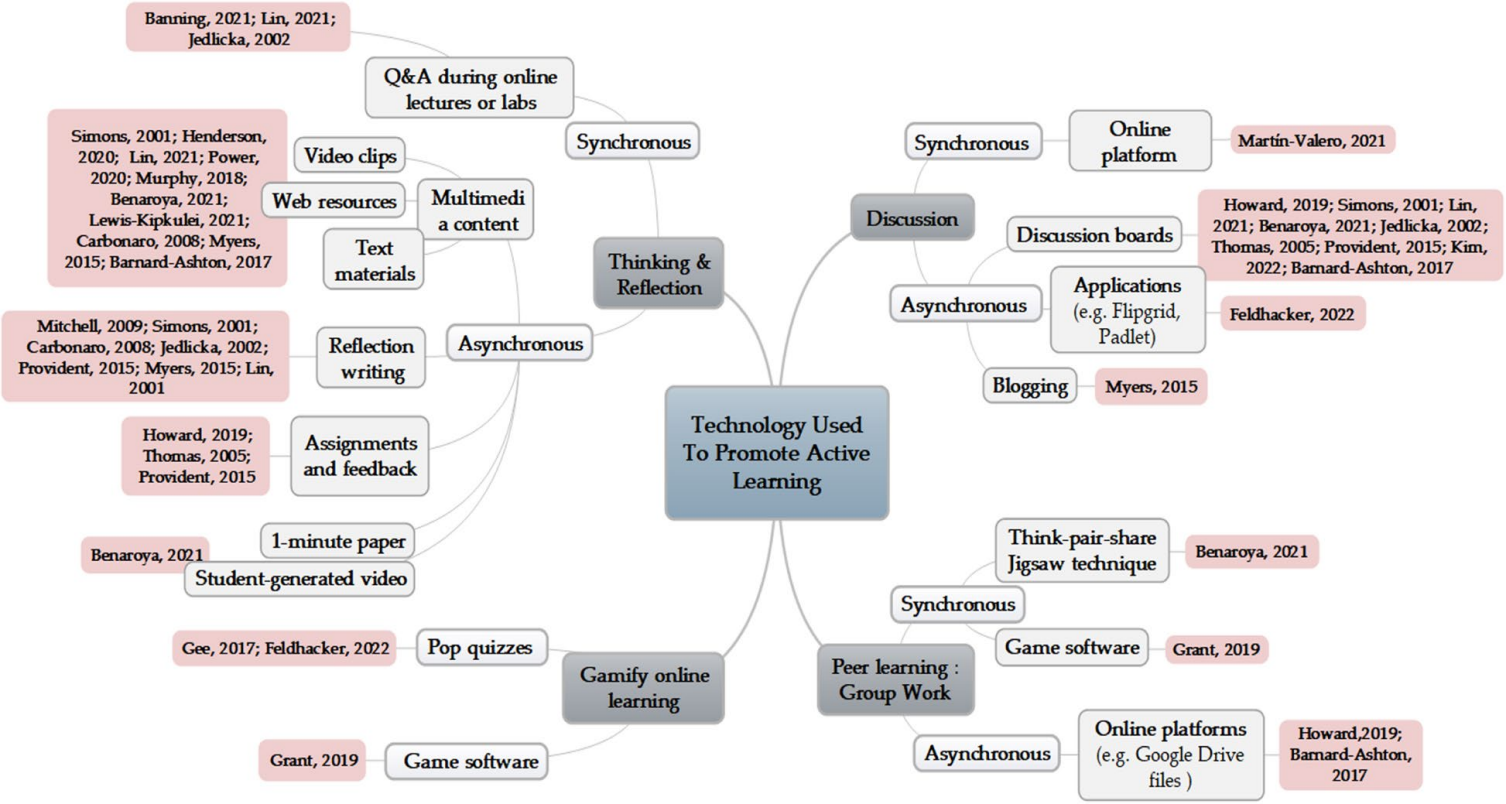
Technologies used to promote active learning

To promote active learning in OT teaching and learning, technologies are largely used for thinking and reflection, discussion, peer learning, and online gamification learning, either synchronously or asynchronously. In thinking and reflection, real-time question and answer during online lectures and labs, multimedia content provision, reflection writing, assignments and feedback, one-minute paper, and student-generated video upload via educational platforms such as LMS were performed. Interactive communication through a platform, discussion boards of the LMS, applications such as flip grids and padlets, and blogging were used. In peer learning, think-pair-share, jigsaw technique activities, and game software were used in real-time, and group activities using online platforms such as Google Drive were conducted. In gamifying online learning, pop quizzes and game software were used (Fig. 3).

## Discussion

This scoping review was conducted to examine digital learning design in OT education, integrated key clinical skills, outcomes, participant experiences, and technologies that promote active learning. The studies included in this review were quantitative, qualitative, and mixed studies to explore digital design in OT education, and most of the studies were observational, qualitative, and mixed studies of these two designs. Additional studies are needed to identify the effectiveness of digital design in OT education. Five major designs have been identified: blended learning, hybrid learning, flipped classroom, and distance learning (including e-learning and MOOC).

### Key clinical skills integrated into digital learning

Professional reasoning is an essential feature of healthcare practice that focuses on assessing needs, planning interventions, and delivering and evaluating health care [47]. Unlike professional reasoning in other fields of health care that focuses on diagnosis or impairment, professional reasoning in OT considers the client, the environment, and the context of performance [39]. For occupational therapists to set treatment goals and outcomes that are appropriate for their clients, they must consider the knowledge, beliefs, environment, and context of their clients and their families [48]. Professional reasoning is the core competency of professional occupational therapists, which is paramount in the client’s problem-solving process and is one of the competencies that must be fostered in the university curriculum. Clinical skills are an important feature of the healthcare professional’s curriculum through specific curriculum hours, a wide range of assessment techniques and procedures, and specially designed laboratories [49]. Clinical skills included physical examination skills, practical procedures, communication skills, and management. Basic scientific knowledge, procedural knowledge, and professional reasoning components are the components for acquiring clinical skills. That is, basic scientific knowledge (declarative knowledge) and procedural knowledge are the underlying knowledge for professional reasoning [17]. Clinical skills in OT include trained basic scientific knowledge to understand the pathophysiology of the client’s impairment, procedural knowledge that serves as a theoretical framework for understanding the client’s problems and solutions, and planning OT intervention strategies. In acquiring these two clinical skills, professional reasoning skills, which is a practical problem-solving process based on case scenarios and related clinical situations, is achieved [50, 51].

Most studies in this review addressed specific subjects and included procedural knowledge with the exception of studies that involved some or all semesters of OT courses. The subjects included procedural knowledge and/or professional reasoning components addressing various OT major courses (e.g., FOR, adaptive equipment, mental health) or interdisciplinary courses (e.g., interprofessional team skills), or case-based professional reasoning training (e.g., sensory processing, OPPM). This shows that professional reasoning, an important competency in OT education, and the procedural knowledge underlying professional reasoning can be integrated into digital learning. One study addressed basic scientific knowledge about human anatomy, and 3D anatomy software was integrated during F2F classes [25]. The 3D software can help understand anatomical relationships beyond the textbook-based 2D format used in the traditional OT curriculum. These results recognize that advanced technology is a useful tool for enhancing basic science knowledge and is becoming a major form of teaching and learning.

### Digital learning designs and outcomes

#### Blended learning

Blended learning involves F2F classes accompanied by online activities and materials. The online materials used were not intended to replace the F2F class session but to supplement the content discussed in the classroom [19]. The blended courses in this review showed similar or greater improvement in learning outcomes compared to the F2F group [25, 26, 31, 39]. This is in line with the blended learning designs having a more effective or equivalent effect than the F2F class on the learning outcome in PT education, as reported by Ødegaard et al. [13]. In addition, students in the blended learning design studies reported subjective opinions such as improvement of communication between students, appropriate course design and online learning environment to achieve learning outcomes and improvements in autonomy and active participation [26–28, 31, 38]. This is consistent with the results of previous studies that framed learning goals; the use of technology to support the achievement of those goals in education improves student engagement, student-student communication, student-instructor communication, and promotes critical discussion [52, 53]. Two studies reported the usefulness of a software tool (3D anatomy and game application for adaptive equipment) used during the F2F class [25, 28]. Game-based learning is becoming an educational technique for reproducing some or all of the clinical experiences in healthcare professional education [54]. In addition, the use of technology such as 3D anatomy has the advantage of increasing students’ motivation to learn and shortening their learning time [55]. Although there is still not enough clear evidence that software is a superior learning tool in OT learning and teaching, it can be a promising tool to enhance clinical skills in OT education.

#### Flipped classroom model

Two studies adopted the flipped learning model and reported a positive impact on learning outcomes in OT education [33, 44]. Ødegaard et al. [13] reported a positive effect of flipped learning on learning outcomes in PT education through a meta-analysis, but Evans et al. [56]. reported that the effect was not clear in healthcare higher education. In OT education, sufficient studies are needed to verify the effectiveness of flipped learning design. The pre-class learning activities of the flipped learning model motivate students, promote participation in learning, and improve the self-regulation, flexibility, and transparency of the learning process. In-class activities help with higher-order thinking by providing opportunities to add new content to existing knowledge to solve problems [57]. These features of flipped design were reflected in the positive experiences of students reported in flipped design studies in this review. They reported positive experiences, such as peer interaction and cooperation, improved participation in learning, and increased individual learning time through the flipped learning design.

#### Hybrid learning

Two of the studies adopted a hybrid learning design. The hybrid and F2F groups showed similar effects on academic outcomes [42], preparation for certification exams, learned skills, and sense of belonging [43]. It was also reported that the number of hybrid course graduates for the state OT association was significantly higher than that for F2F [43]. This means that hybrid learning is a learning method with the potential to reach a wider audience by allowing access to education anywhere as well as equality of learning outcomes. In a review by Raes et al. [58], participants reported that hybrid learning is flexible in students’ course attendance in higher education, and creates richer learning experiences through collaboration and connections between F2F and distant students, thus facilitating students’ exposure to broader perspectives and ideas. This is consistent with the results of hybrid learning included in this review. However, there are challenges in the design and implementation of educational strategies and technical systems that are suitable for hybrid learning [58]. In addition, there is insufficient research to discuss the effectiveness of hybrid learning and students’ experiences in OT education. In future OT education, more research is needed on various educational scenarios for hybrid learning and their effect on learning outcomes.

#### Distance learning; E-learning, MOOC

Distance learning is the most important phenomenon in higher education today [59], providing learners with flexibility, mobility, and choice for learning [60]. E-learning was the most common distance learning method identified in this review (*n* = 10), and one study adopted the MOOC [32].

Distance learning has become an increasingly important mode of learning and teaching in conventional universities, continuing education, and corporate training [61]. The features of distance learning, including these various learners, are discussed in this review. Distance learning studies included a variety of learners, including fieldwork education and continuing education for clinicians, as well as campus classes for OT students. Compared with traditional learning, distance learning in professional healthcare education has reported similar or small positive effects on professional knowledge, attitudes, skills, and satisfaction [62, 63]. The results of distance learning in this review are also consistent with those of previous studies. Compared with F2F, the distance learning course showed similar or greater improvement in academic performance [29, 30, 32, 37]. The pivotal role of e-learning is interaction and practice exercises, repetition, and feedback, which are related to improving learning outcomes [64]. This is consistent with the perceptions of students’ experiences. The roles of e-learning were implemented with various web-based tools, including the virtual classroom platform, LMS, and experiences such as interaction and learning autonomy and promotion of higher-order thinking. However, some students commented on the pedagogical and technical challenges of e-learning. They reported that they preferred the combination of e-learning and F2F [29] and that e-learning has limitations in interaction between learners [37], unlike F2F. Technology is a physical tool, not a theoretical thinking tool or concept. However, it changes the way we think about tasks and how we perform them [65]. Therefore, in e-learning, it is necessary to plan an appropriate learning design for the teaching and learning platform in consideration of the characteristics of the subject (e.g., theory class/practical class). In addition, setting up an appropriate online environment for active learning, such as interactions between learners, and equipping instructors and learners with competence in using the technology will be important aspects.

#### Digital learning promotes active learning

Active learning strategies are applied to online sessions for various reasons. The purpose of active learning is to engage learners in higher-order thinking (e.g., analysis, synthesis, and evaluation) that enables them to assimilate, apply, and sustain learning [66]. It also accommodates learners’ diverse learning styles, promotes learner achievement, strengthens motivation, and enables them to learn more [66]. Most of the studies in this review applied various active learning strategies, either synchronously or asynchronously, such as thinking and reflection, discussion, peer learning-group activity, and gamifying online learning through various contents delivered electronically. An online session involves the continuum of content delivered electronically, from single assignments [67], and the use of computer-based learning management systems to fully web-based courses [68]. In particular, nine studies in this review used management system platforms such as Blackboard, WebCT (web courset Tools), and Moodle [26, 27, 31, 34–36, 41, 45, 46], most of which have been applied in e-learning course design. The core functions of the system are student management and tracking, material presentation, communication, scheduling, and learner testing. These systems focus on collaboration between learners and instructor feedback through discussion forums and student e-projects. It is therefore well-suited for engaging learners in active learning strategies, which are active processes that allow instructors and learners to become knowledge-building partners [69]. Online sessions of blended, flipped, and hybrid learning also played a leading role in integrating active learning with effective learning activities, such as discussions, project-based or problem-based assignments, or laboratory exercises. Students who participated in these courses reported that active learning strategies could provide immediate and frequent feedback from instructors during active learning activities and facilitate collaboration and interaction with other students. In addition, students reported that it helped them to have a broader understanding of the learning content and build their own learning style. This implies that, in OT education, an online format can be an effective means of acquiring knowledge and skills by integrating active learning strategies. In addition, it is necessary to plan a learning design that considers the effective active learning strategies that can be incorporated into the online format.

### Limitations

Due to the nature of the scoping review, which aims to provide an overview or map of the evidence, this study did not evaluate the risk of bias, so it was not possible to clarify the reporting, methodological quality, and intervention effectiveness of the included studies. Some studies showed a lack of detail in interventions, while others reported non-validated outcome measures such as self-reports and underreported statistical methods. It is difficult to evaluate the effectiveness of different designs in digital learning because of the limited number of studies in each digital learning design, and many of the studies included in this review involved qualitative analyses of students’ perceptions of learning experiences. However, we found positive responses to the design of digital learning that included improved academic performance, professional reasoning, learning participation and satisfaction, active learning, self-confidence, and overall efficiency of learning. Although the formation of the research question and search process was systematically conducted for a high level of scientific quality, the search strategy and exclusion criteria may have resulted in the omission of related studies. In addition, although various types of digital learning designs were identified and analyzed in this review, the definitions used may not be complete, and thus, there may be limitations in comparing designs and synthesizing the results. Finally, although this analysis of this clinical skill type may be framed as outside this scoping review, this is not the main purpose or intent of this study. In the future, it will be necessary to demonstrate outcomes, including the effectiveness of digital learning, using more robust study designs and experimental studies. With the rapid evolution of the use of technology in learning and the expanding associated literature, it is imperative that the definition and division of digital learning are clarified.

## Conclusions

This review identified the digital learning designs applied in OT teaching and learning. The digital learning designs identified in this review were flipped learning, blended learning, hybrid learning, and distance learning, including e-learning and MOOC. Among the components of clinical skills, professional reasoning is the core competency of professional occupational therapists, and procedural knowledge is the main knowledge to acquire. This review has shown that these components of clinical skills can be integrated into digital learning in OT education. Digital learning designs applied to OT education have many benefits. This includes improving the learning outcomes of knowledge and skill acquisition, enhancing learning participation and reflection, and collaboration between learners. In addition, various technologies used in digital learning facilitate active learning by providing learning strategies, such as thinking and reflection, discussion, peer learning-group activity, and gamifying online learning, either synchronously or asynchronously. Although digital learning designs have had a positive impact on OT education, the results are limited to the OT population included in this review. Therefore, it is necessary to confirm the results of future studies with larger experimental designs. In addition, some studies have reported minimal barriers to digital learning. This review suggests a need for digital learning design plans that consider learning subjects and appropriate technologies for effective learning.

## Data Availability

The data that support the findings of this study are available from the corresponding author, upon reasonable request.
